# Practical actions of nurses working at community general support centers in Japan: A qualitative study

**DOI:** 10.20407/fmj.2025-012

**Published:** 2026-02-28

**Authors:** Miho Miyamoto, Rumi Seko

**Affiliations:** Faculty of Nursing, Fujita Health University, School of Health Sciences, Toyoake, Aichi, Japan

**Keywords:** Community general support center, Community nurse, Competency, Older adults, Preventive care

## Abstract

**Objective:**

To clarify the practical actions that experienced nurses working at community general support centers perform to provide comprehensive community care that supports the lifestyles of residents, including older adults.

**Methods:**

The participants were full-time nurses with at least 9 years of experience working at community general support centers in Japan. Semi-structured in-person interviews were conducted between August and December 2023, and the results underwent qualitative descriptive analysis.

**Results:**

Ten core categories, twenty-six categories, and eighty-eight subcategories were identified as actions that nurses at community general support centers performed. The 10 core categories were assessment for care prevention, communication that builds trust, networking, promotion of nursing care prevention, coordination of the care team, creation of projects and resources that can be locally implemented, creation of a local care system, teamwork among professions at the community general support center, self-improvement to improve one’s expertise, and operational management for implementing effective support.

**Conclusions:**

Nurses performed practical actions to both provide individual support and community development. The results suggested that nurses used the networks they had built through performing their daily work duties, including providing support to individuals, to support community resources. They also utilized community resources in local communities to create care systems in the community.

## Introduction

As authorized by the Long-Term Care Insurance system in Japan, community general support centers act as key facilities in providing comprehensive care at the level of the community. They do so by supporting the lifestyles of residents, including older adults. General consultations, protection of rights, comprehensive and continuous care management, and management of nurse-administered preventive care are carried out at these centers.^1^ Facilities may be directly managed by local municipalities or managed by commissioned private corporations. These facilities are staffed by public health nurses, social workers, and senior care managers, but securing personnel is an issue. When securing sufficient public health nurses proves challenging, a nurse may be considered a public health nurse if they fulfill certain criteria. These criteria include, amongst others, having experience in community care and community nursing as well as having at least 1 year of experience in public health nursing related to care for older adults. Nurses who fulfill these criteria may be posted to community general support centers; this happens frequently at facilities with commissioned management.^2^ Therefore, nurses at community general support centers perform the work of a public health nurse and they are considered to be equivalent to such. However, these nurses face difficulties, such as a workload that carries a heavy quantitative and qualitative burden, as well as a lack of ability, such as not knowing how to encourage the building of a community.^2,3^ Consequently, cultivating human resources and retaining personnel have become issues.^2^

Recently, the concept of competencies has been introduced to the cultivation of human resources in various fields. Spencer and Spencer developed the concept of competency by gathering qualitative competency data from interviews conducted with high performers.^4^ Previous studies in Japan have described competencies for government public health^5,6^ and hospital^7,8^ nurses, while previous studies involving community general support centers have described performance indicators for public health nurses.^9–12^ However, because no studies have focused on nurses and most studies targeted both public health nurses and nurses as participants, the status of the practical actions that nurses are performing at community general support centers remains unclear.

To qualify as a public health nurse in Japan, one must have a basic qualification in nursing before studying community nursing as well as public health and welfare administration. When work duties target a community, the person performing them must do so with a public health nurse qualification as foundation. Public health nurses at community general support centers are expected to identify community issues based on their experiences of treating individuals, and then use this knowledge to create a community in which nurse-administered preventive care is encouraged. To achieve this, clarifying the practical actions that nurses perform in their role as public health nurses in community general support centers, investigating occupational characteristics, and securing human resources to perform work duties going forward while also considering on-the-job training is important.

Therefore, the purpose of the present study was to clarify the practical actions of nurses working at community general support centers. An interview survey was used to determine the conscious practices of experienced nurses working at a such centers that enable them to provide high quality support to older adults. The *high quality support to older adults* referred to in this study was defined as support that enables frail and or older adults to autonomously and positively engage in their daily lives with support and care from their family, neighbors, and specialists, as well as the building of organic frameworks that enable community residents, including older adults, to live with peace of mind even as they age. The term *experienced nurses* referred to nurses with a wide range of experience working at a community general support center together with experience in providing high quality support to older adults.

## Methods

### Participants

The participants included in this qualitative descriptive study were nurses working full-time at community general support centers in Aichi Prefecture, Japan. Nurses who had a wide range of work experience as well as practical experience in providing high quality support to older adults were included. We investigated the number of years required for being referred to as having a wide range of work experience as follows: Using a fact-finding survey^13^ as reference, we determined that 5 years of experience of being in charge of many different work duties related to both providing individual support and also building a community was valid. The establishment of community general support centers began in 2006; therefore, at the time of the survey, the greatest possible number of years working at such a center was approximately 15 years. However, we set the criterion that at least 6 years of experience were required because staff may have been transferred to other departments, thus reducing the number of years of continuous experience. Using the recruitment method, we explained an outline of this study to all facility managers in the prefecture. The managers then referred 10 participants with at least 9 years of experience working at a community general support center to the authors.

### Data collection

Based on an interview guide, semi-structured interviews were conducted individually with each participant in person between August and December 2023. Prior to conducting the interviews, the definition of high quality support to older adults used in this study was shared between the participants and interviewers. During the interviews, participants described cases in which they had provided high quality support to older adults. They were asked about actions and aims when providing such support as well as what they kept in mind when providing high quality care and their aims regarding such. Interviews were recorded with the participants’ consent. The basic characteristics that were asked were age, sex, number of years of experience as a nurse, and number of years of experience working at a community general support center.

### Data analysis

Data underwent qualitative, descriptive analysis. The interviews were transcribed and then read again to extract phrases participants used to describe what kind of actions were performed with what kind of aims and in what kind of practical situations. Words best describing meaningful actions were utilized to create summaries that were then codified. Similar action codes were grouped together, and subcategories were identified, ensuring that relationships amongst shared actions and their aims were preserved. Similar subcategories were grouped together to identify categories. Then, to extract core categories, we increased the level of abstraction while considering the objectives of category actions.

### Ethical considerations

The purpose of the study was appropriately explained to participants and facility managers; that participation was based on free will and that anonymity would be preserved were also appropriately explained to them. Participants confirmed their consent by signing the consent form. This study was approved by the institutional review board of our institution (approval number HM22-445).

## Results

### Summary of participants and affiliated institutions

Ten participants were included: Their characteristics are shown in Table 1. The participants ranged in age from being in their 40s to being in their 60s; all were female. All the nine affiliated institutions, included from seven small- to medium-sized cities, were managed by commission. On average, the participants had worked at a community general support center for 12.8 years. The interviews lasted for 52 to 101 minutes.

### Practical actions of experienced nurses working at a community general support center

Analysis of the 455 codes describing actions performed when providing high quality care for older adults revealed 88 subcategories, 26 categories, and 10 core categories. Table 2 shows the core categories and categories; the findings are organized below according to core category. Categories are marked with << >> and subcategories with < >.

#### (1) Assessment for care prevention

Comprising three categories, this core category described actions involving the assessment of the current state of residents, including older adults, using multifaceted information gathering to prevent nursing care. In terms of individual support, nurses travelled to the residence of the older adult to <<observe and listen to their actual life circumstances from a wide range of perspectives to gain understanding of the actual living conditions and assess the lifestyle desired by the older adult and their families from the perspective of nursing care prevention>>. The specific actions that nurses performed were assessing the older adult’s strength and lifestyle issues, as well as assessing future risks. Nurses would also offer support from a perspective related to treatment and life such as <conducting a physical, medical assessment of the older adult and determining whether medical care was necessary> based on their clinical experience of working at a hospital. Nurses would also cumulatively <<assess local issues and needs based on local conditions>> identified in daily work duties and <<have a vision for feasible regional development that made use of the characteristics of the region>> while trying to identify the best direction forward for the local community and how the nurse could be of help to the local community.

#### (2) Communication that builds trust

This core category described actions involving communication aimed at cultivating relationships of trust with those who received individual support and their families, as well as with various others, including residents and related parties. Three categories formed this core category: With the aim of <<promoting mutual understanding through dialogue>> with various people in the community, nurses first strove to <<listen carefully to the older adult and residents and understand them>> before <<raising awareness of the community general support center>> in order that the nurse’s activities were understood. As reflected by Participant F’s statement, “I am careful to build good relationships because, if I do not, it will impact various areas of our activities,” participants valued <engaging in communication that encourages reliance> and <sincerely responding to questions and requests>.

#### (3) Networking

The core category of networking described actions involving creating networks to gather information useful for supporting older adults and comprised three categories. Participant D’s statement, “They (networks) are the underlying support for all that we do,” reflects the sense that participants had that the networks they had built formed the basis of their practical actions. During their daily practice, nurses would <go out into communities and talk to many different people> and offer consultations, thereby <<raising awareness of the community general support center and creating opportunities for connections>>. Nurses would then <exchange information and establish relationships for further information exchange> with people with whom they had connected, <<creating routes to connect with information>>. Furthermore, they focused on <creating win-win relationships through information exchange and offering consultations> in these relationships and were <<building a network by accumulating routes to connect with information>> while maintaining and expanding these beneficial relationships year-by-year.

#### (4) Promotion of nursing care prevention

This core category, comprising two categories, described actions that involved promoting nursing care prevention for older adults and residents with an awareness of acting as a leader in nursing care prevention in the local community. In individual support, nurses would <help older adults to reflect on their daily lifestyle through conversation> to promote the need for nursing care prevention and <slowly introduce interventions while confirming the intentions of the older adult>, <<leading older adults to adopt behavioral changes to prevent the need for nursing care>>. In local communities, nurses were involved in <<promoting communities for nursing care prevention>> by offering information about preventing nursing care and <offering easy-to-understand explanations based on prior case examples and effects related to community-building with the aim of nursing care prevention among residents>.

#### (5) Coordination of the care team

Composed of two categories, this core category described actions that involved coordination to enhance cooperation amongst care teams that provided support to older adults. First, to construct a care team, nurses would <<find and connect with people who could provide support and local social resources>> while <using networks that they had cultivated to search for means of support>. Then, they would <coordinate roles based on an understanding of the position of each supporter and related facility>. As evidenced by the statement of Participant I, “I believe that my role in filling the gap between hospital and home care is offering specialized medical knowledge,” nurses also acted as a bridge between medical and nursing care because they <facilitated coordination between medical and welfare professionals>. By doing so, nurses would <<coordinate to increase cooperation among those who support older adults>>.

#### (6) Creation of projects and resources that can be locally implemented

With two categories, this core category described actions involving the development of projects to enable local communities to hold exercise workshops and groups et cetera. Nurses <consulted with residents and related facilities about ideas for projects and asked for their opinions>, using their networks to task members to set up such projects. To make such projects operational, “While discerning what they (residents) want to do and what the level of their motivation is … (Participant C),” nurses were <identifying the operational status of the facility and residents who could be involved in the project and supporting them so that they could continue with such projects> to <<plan local projects and promote their launch and operation>>. Nurses were also engaged in <<discovering resources not locally available and looking ahead to developing new resources>> by <evaluating current community projects and considering possibilities for the development of new projects in response to unresolved issues>.

#### (7) Creation of a local care system

This core category described actions that helped communities to solve community problems within the community with to support the needs of older adults in local communities, and was composed of three categories. Nurses were involved in <gathering information on the status of local communities in response to community issues and explaining these issues to residents at, amongst others, community care conferences> as well as <actively proposing issues of concern and worries at conferences> to first <<share local issues that need to be resolved with residents and people connected to the area>>. Participant F’s statement, “I previously matched some needs by connecting a person who had an idea that they wanted to try implementing in the community with a dementia café that was being planned,” illustrates how nurses were involved in <<proposing solutions to local issues>> by engaging in actions such as <using networks that they had cultivated to match needs with community needs>. This statement by Participant C, “Even if the community resources exist, people cannot use them if they do not know about them, so I type them out into a table and present them at regular Municipal Welfare Commissioners’ Association meetings and local resident meetings,” shows how nurses <work to enable the local utilization of community social resources> and <regularly hold collaborative meetings with residents and related parties to create opportunities for dialogue> to engage in actions to <<work to increase cooperation between residents, related parties, and relevant organizations in the region>>.

#### (8) Teamwork among professions at the community general support center

Comprising three categories, this core category described actions to enhance teamwork within the community general support center. Nurses <daily engaged in reporting, correspondence, and consultations to constantly share information amongst staff at the community general support center> and <constantly discussed cases of support methods amongst staff of the community general support center>. Thus, they valued and aimed to <<create a system that can handle any type of job at the community general support center>>. By <teaching each other specialized knowledge and skills amongst the various types of occupations at the community general support center>, they <<effectively utilized the expertise of each community general support center>>. To <<improve relationships between professions at the community general support center>>, nurses engaged in actions such as suppressing their feelings to preserve relationships with people of other professions when performing actions that would demonstrate their expertise as a nurse, such as <adjusting their awareness of their role as a nurse>.

#### (9) Self-improvement to improve one’s expertise

This core category described actions involving managing oneself to offer support in accordance with functional roles of the community general support center. It was composed of three categories. With the aim of <<improving support skills as a staff member at the community general support center>>, nurses <enhanced their facilitation skills so that they were able to utilize them during discussions> and <made efforts to convey information in a way that would be understood by the listener>. Nurses <<updated the knowledge and information necessary to carry out works>> by <gathering information on initiatives in neighboring municipalities, as well as information and reports by the government and prefectures>. Participant D’s statement, “I enjoy connecting with various people,” affirms that nurses were <<enjoying working with residents>>.

#### (10) Operational management for implementing effective support

This core category, comprising two categories, described actions involving operational management to implement effective support at the community general support center. To <<carry out the works in a planned manner>>, nurses would organize their duties to guarantee quality and complete activities as soon as possible. Because they were required to <make decisions and act quickly when faced with an emergency>, nurses were careful to <<make quick decisions and prioritize works>>.

## Discussion

### Features of practical actions of nurses at a community general support center

Of the ten core categories of actions identified in this study, those of assessment for care prevention, communication that builds trust, networking, promotion of nursing care prevention, care team coordination, creation of projects and resources that can be locally implemented, and creation of a local care system supported older adults and local communities in preventing the need for nursing care. The core categories of teamwork among professions at the community general support center, self-improvement to improve one’s expertise, and operational management for implementing effective support appeared to be actions comprising their response as a member of the community general support center. Three features were noted in the practical actions of community general support center nurses.

The first feature was the fact that nurses leveraged skills gained from their clinical experience at a hospital to provide support in coordinating residents and related parties. These findings indicated actions involving decision-making on the timing of medical interventions, depending on the older adults’ need for medical care and the state of their disease, and the actions of <facilitated coordination between medical and welfare professionals>. Although the coordination of home visits to provide medical and nursing care and dementia countermeasures is implemented to build community general care systems, community general care center nurses could be expected to play a role in offering appropriate responses to life and forming a bridge between medical and nursing care.

The second feature was that nurses acted from the perspective of providing individual support and community development. These actions were similar to those performed by public health nurses at community general support centers.^9–12^ We found that nurses used networks they had developed in daily practice, such as providing individual support, to find and connect with people who can provide support and local social resources. Nurses coordinated with various people in the community, including social workers, residents, and related institutions, to offer support that was adapted to utilize the available resources that the community could provide. The basis for acting to support residents according to the circumstances of each community appeared to result in support being offered by both individuals and communities.

The third feature was that, to provide support on a community level, nurses aimed to build community care systems by expanding on the methods of support developed when providing individual support. The findings indicated that, when supporting older adults, nurses exchanged information with residents and related facilities while increasing the number of companions available to provide support to older adults and repeating this process to engage in community networking. Nurses used the networks that they had cultivated to match needs with community needs and lobbied to allow residents and supporters access to community resources. Miwa and Kono found that nurses engaged in community care might discover health issues because their perspective was different to that of public health nurses employed by local governments and might create community activities.^14^ Our findings indicated that, while exploring supportive methods for the health issues of individual patients, nurses identified new community resources and leveraged these to provide support, which created opportunities to develop community resources. By enabling other patients and people involved in providing support to use these coordinated community resources, nurses promoted the utilization of community resources in local communities. These actions of nurses at community general support centers could be a means of building community care systems.

### Limitations and future challenges

This study had several limitations. First, because all ten participants were working at nine facilities in seven small- to medium-sized cities with a low average rate of aging and all within the same prefecture, the target region and sample size were limited; this created bias. According to a fact-finding survey of community general support centers throughout Japan, centers in large cities with populations of 500,000 people or more reported working long hours on general consultation duties whereas towns and villages with populations of fewer than 50,000 people reported working long hours on providing dementia support and general nursing prevention support.^13^ Because work duties differ depending on the scale of the population, rates of aging, and regional characteristics and more, this may have impacted the data. Second, the methodology was limited: This study used a qualitative descriptive design to extensively examine nurses’ practical actions. Specific practical actions that nurses perform were identified and characteristic actions suggested, indicating that the findings have a certain significance. However, the possibility that the qualitative analysis impacted the findings must be acknowledged. For future research, a large-scale verification of content validity that includes use of the Delphi method must be conducted.

## Figures and Tables

**Table 1:** Overview of study participants and their affiliated institutions

	A	B	C	D	E	F	G	H	I	J
Age	50s	60s	50s	50s	50s	50s	60s	40s	50s	50s
Years of experience as a nurse	15	20	34	20	30	36	50	20	21	30
Mean±Standard deviation	25.2±12.5									
Years of experience at community general support center	12	11	17	10	13	15	14	9	11	16
Mean±Standard deviation	12.8±2.5									
Facility management	Commissioned management	Commissioned management	Commissioned management	Commissioned management	Commissioned management	Commissioned management	Commissioned management	Commissioned management	Commissioned management	Commissioned management
Municipality	City Z	City Y	City X	City W	City X	City X	City V	City V	City U	City T
Population size of municipality	Medium city^2^	Core city^1^	Small city^3^	Medium city^2^	Small city^3^	Small city^3^	Medium city^2^	Medium city^2^	Small city^3^	Medium city^2^
Aging rate of municipality	Approx. 25％	Approx. 24％	Approx. 27％	Approx. 20％	Approx. 27％	Approx. 27％	Approx. 26％	Approx. 26％	Approx. 26％	Approx. 27％

1) Core city: Population over 200,000 2) Medium city: Population over 100,000 3) Small city: Population below 100,000

**Table 2:** Categories for the practical actions of nurses at community general support center

Core category	Category
Assessment for care prevention	observe and listen to their actual life circumstances from a wide range of perspectives to gain understanding of the actual living conditions and assess the lifestyle desired by the older adult and their families from the perspective of nursing care prevention
	Assess local issues and needs based on local conditions
	Have a vision for feasible regional development that makes use of the characteristics of the region
Communication that builds trust	Listen carefully to the older adult and residents and understand them
	Raising awareness of the community general support center
	Promoting mutual understanding through dialogue
Networking	Raising awareness of the community general support center and creating opportunities for connections
	Creating routes to connect with information
	Building a network by accumulating routes to connect with information
Promotion of nursing care prevention	Leading older adults to adopt behavioral changes to prevent the need for nursing care
	Promoting communities for nursing care prevention
Coordination of the care team	Find and connect with people who can provide support and local social resources
	Coordinate to increase cooperation among those who support older adults
Creation of projects and resources that can be locally implemented	Plan local projects and promote their launch and operation
	Discovering resources not locally available and looking ahead to developing new resources
Creation of a local care system	Share local issues that need to be resolved with residents and people connected to the area
	Proposing solutions to local issues
	Work to increase cooperation between residents, related parties, and relevant organizations in the region
Teamwork among professions at the community general support center	Create a system that can handle any type of job at the community general support center
	Effectively utilize the expertise of each community general support center
	Improve relationships between professions at the community general support center
Self-improvement to improve one’s expertise	Improving support skills as a staff member at the community general support center
	Update the knowledge and information necessary to carry out works
	Enjoying working with residents
Operational management for implementing effective support	Carry out the works in a planned manner
	Make quick decisions and prioritize works

## Availability of Data and Materials

All data generated or analyzed during this study are included in the article.

## Supplementary Material

PDF-Japanese
