# Supplementary material for: Practical actions of nurses working at community general support centers in Japan: A qualitative study
Source: Fujita Med J. 2026 Feb 28;12(2):129–34. doi: 10.20407/fmj.2025-012 (PMC13129712; doi:10.20407/fmj.2025-012)
Supplement: Supplementary file 1 — PDF-Japanese [file fmj-12-129_s1.pdf]

Abstract

目的：高齢者を含む住民の生活支援をすることにより地域包括ケアを目指す地域包括支援センターで働く経験豊かな看護師の実践行動を明らかにする．

方法：研究参加者は，地域包括支援センターで 9 年以上の経験をもつ常勤看護師とした．半構造化面接を行い，質的記述的に分析した．

結果： 地域包括支援センター看護師の行動として，【介護予防に向けたアセスメント】，【信頼関係を育むコミュニケーション】，【ネットワークづくり】，【介護予防の牽引】，【ケアチームのコーディネーション】，【地域で実践できる事業・資源の創設】，【地域のケアシステムづくり】，【地域包括支援センター職種間のチームワーク】，【専門性を高めるための自己研鑽】，【効果的な実践をするための業務マネジメント】の 10 コアカテゴリー，26 カテゴリー，88 サブカテゴリーが抽出された．

結論：看護師は，個別支援と地域づくりの両者の視点を持って実践していた．個別支援などの日常業務で培ったネットワークを活かして，地域の資源を支援につなげていた．さらに，地域の資源を地域で活用できるように働きかけることで，地域のケアシステムづくりを目指そうとする行動が示唆された．

Key words：Community general support center, Community nurse, Competency, Older adults, Preventive care

序論

地域包括支援センターは，介護保険法で位置づけられた高齢者を含む住民の生活を支援することにより，地域包括ケア実現に向けた中核的な機関で，総合相談，権利擁護，包括的・継続的ケアマネジメント，介護予防ケアマネジメント等の機能を担っている[1]．施設運営は，市町村が運営する直営型と民間企業が運営する委託型があり，職員体制は，保健師，社会福祉士，主任介護支援専門員の 3 職種が配置されている．しかし，現場では職員確保の課題があり，保健師確保が困難な場合には，地域ケア，地域保健等に関する経験および高齢者に関する公衆衛生業務経験を 1 年以上有する看護師を保健師に準ずる者と規定し，前述の条件を充たした看護師が配置されている施設もあり，特に委託型の施設に多い[2]．そのため，地域包括支援センターの看護師は保健師に準ずる者として，保健師の職務を遂行している．一方，業務が量的質的に負担となっていることや地域づくりをどう進めてよいかわからないなどの力量不足の困難感が報告されており[2,3]，職員の人材育成や職員の定着が課題とされている[2]．

近年, 様々な職務の人材育成においてコンピテンシーの概念が導入され, Spencer ら（2011）は，高い業績者へのインタビュー調査等より質的にコンピテンシー項目を収集し，コンピテンシーを開発した[4]．国内の先行研究では、行政保健師のコンピテンシー[5,6]や病院看護師のコンピテンシー[7,8]等が報告されている．地域包括支援センターに関する先行研究では，保健師の活動指標[9-12]などは報告されているが，看護師に焦点化された報告はみられず，多くは保健師と看護師を合わせて対象者としているため，地域包括支援センター看護師の実践活動が明らかでない状況である．日本の保健師資格は，看護師資格を基礎資格として，地域看護と保

健福祉行政をさらに修得している．地域を対象として行う業務には，保健師資格に基づいた視点が欠かせず，地域包括支援センター保健師の役割として，個への対応から地域の課題を見出し，介護予防の地域づくりへ展開していくことが期待されている．そのため，地域包括支援センター保健師の役割を担っている看護師の実践行動を明らかにし，職種の特徴を検討することは，今後の業務に応じた人材確保や現任研修を考えるうえで重要と考える．

そこで本研究の目的は，経験豊かな地域包括支援センター看護師が質の高い高齢者支援を実践するために意識している行動のインタビュー調査により，地域包括支援センターで働く看護師の実践行動を明らかにすることである．本研究において「質の高い高齢者支援」とは，高齢者あるいは虚弱高齢者が，家族，近隣住民，専門職の支援や見守りを得ながら，自立的に，前向きに生活できるように支援すること，および，高齢者を含む地域住民が高齢になっても安心して生活できる有機的な仕組みをつくることとした．「経験豊かな看護師」は，地域包括支援センターの業務を幅広く経験し、質の高い高齢者支援の実践経験がある者とした．

研究方法

研究参加者

愛知県内の施設に所属している常勤の看護師で，幅広い業務経験を有し，かつ質の高い高齢者支援の実践経験がある者とした．幅広い業務経験がある年数の設定は，以下の検討をした．実態調査[13]を参考に，個別支援のみでなく地域づくりの業務も多く担っている経験 5 年以上が妥当と考えられた．一方地域包括支援センターは 2006 年より設置が開始され，調査時

点の最長経験年数は約 15 年であったが，他部署への移動があるため継続年数が短い可能性があることを考え、6 年以上の者とした．リクルート方法は，県内全ての施設管理者に本研究の主旨を説明し，地域包括支援センターの経験が 9 年以上の 10 人の紹介を得た．

データ収集方法

インタビューガイドに基づき，対面にて半構造化面接による個別インタビューを 2023 年 8～12 月に行った．インタビュー開始前に参加者とインタビュアーとで本研究における「質の高い高齢者支援」の定義を共有した．インタビュー内容は，質の高い高齢者支援であった事例を語ってもらい，その支援で行った行動とその意図，また質の高い実践のために意識して行っていることとその意図を尋ねた．インタビュー内容は参加者の同意を得て録音した．基本属性として，年齢，性別，看護師の経験年数，地域包括支援センターの勤務経験年数を尋ねた．

データ分析方法

データは，質的記述的方法で分析した．インタビュー内容を逐語録に起こして繰り返し読み，参加者が「どのような実践場面で，どのような意図でどう行動したのか」を表現している言説を取り出し，意味する行動を最も表現している言葉を活かして要約しコード化した．類似した行動のコードを集約し，共通する行動とその意図の関係性を損なわないように，サブカテゴリーを抽出し，サブカテゴリーの共通性から集約しカテゴリーを抽出した．さらに，カテゴリーの行動のねらいを考慮しながら抽象度を高めコアカテゴリーを抽出した．

倫理的配慮

研究参加者および施設管理者に，本研究の趣旨および参加の任意性と匿名性の保持について十分説明し，同意書への署名をもって同意を確認した．なお，本研究は藤田医科大学医学研究倫理審査委員会の承認を得て実施した（承認番号：HM22-445）．

研究結果

研究参加者および所属施設の概要

研究参加者は 10 人で，属性を表 1 に示す．年齢は 40～60 歳代で，全員女性であった．所属施設は中小都市部の 7 市にある 9 施設で，運営形態は全て委託型で，地域包括支援センターの平均経験年数は 12.8 年であった．インタビュー時間は，52～101 分であった．

経験豊かな地域包括支援センター看護師の実践行動

質の高い高齢者支援を実践するための行動として得られた 455 コードを分析した結果，サブカテゴリー88，カテゴリー26，10 のコアカテゴリーが抽出された．表 2 にコアカテゴリーとカテゴリーを示した．以下，コアカテゴリーごとに結果を示す．なお，コアカテゴリー【 】，カテゴリー《 》，サブカテゴリー〈 〉，研究参加者の語りは「*斜体*」で表記した．

（1）【介護予防に向けたアセスメント】

このコアカテゴリーは，高齢者を含む地域住民を多面的に情報収集し介護予防の視点で現状をアセスメントする行動で，3 つのカテゴリーから構成された．個別支援として，高齢者の生活の場に出向いて《幅広い視点から実際の生活を見聞きして生活実態を把握し，高齢者と家族が望む生活を介護予防の視点でアセスメントする》ことをしていた．具体的な行動では，

高齢者の力量や生活課題の背景，今後のリスクなどをアセスメントしていた．また，病院の臨床経験を踏まえて〈高齢者の身体面を医学的にアセスメントして医療の必要性を判断する〉など治療や生命に関わる視点からも対応していた．一方，日々の業務で把握した《地域の状況から，地域の課題やニーズをアセスメントする》ことを蓄積し，どのような地域になったらよいか，この地域で何ができそうかなどを模索しながら《地域の特性を生かした実現可能な地域づくりのビジョンを持つ》ことをしていた．

(2)【信頼関係を育むコミュニケーション】

このコアカテゴリーは，個別支援で関わる対象者と家族だけでなく、住民や関係者など関わった様々な人と信頼関係を育むことを目指したコミュニケーション行動で，3 つのカテゴリーから構成された．地域のあらゆる人と《対話をとおして相互理解を図る》ことを目指して，先ず《高齢者や住民の話を傾聴して相手を理解する》ことを行い，自分たちを理解してもらうために《地域包括支援センターを知ってもらう》行動をしていた．研究参加者の *「上手く関係づくりをしていかないと，いろんな所に影響がでてくるので，丁寧にと思ってます(F 氏)」* との語りのように，〈頼ってもらえるようなコミュニケーションをとる〉ことや，〈相手の相談や依頼に誠実に対応する〉ことを大切にしていた．

(3)【ネットワークづくり】

このコアカテゴリーは，高齢者支援を行うための情報が入るネットワークをつくる行動で，3 つのカテゴリーから構成された．研究参加者は，*「それ（ネットワーク）で支えられている(D 氏)」* との語りのように，自身が培ってきたネットワークが実践の基盤と捉えていた．看

護師は，日頃から〈地域に出向いて，いろんな人に会って声をかけて話をする〉ことや，相談を持ちかけたりすることで,《地域包括支援センターを知ってもらい繋がりのきっかけをつくる》行動をしていた．その繋がった人と〈情報交換を通じて，情報が行き来きできる関係性をつくる〉ことで《情報とつながるルートをつくる》ことをしていた．さらにこの関係性は，〈情報交換や相談対応を通じて，ウィンウィンな関係をつくる〉ことを意識して，このいい関係性を年単位で維持し広げながら《情報とつながるルートを積み重ねてネットワークをつくる》ことをしていた．

（4）【介護予防の牽引】

このコアカテゴリーは，地域の介護予防の牽引役という意識を持って、高齢者や住民に介護予防を働きかける行動で，2 つのカテゴリーで構成された．個別支援では，高齢者との〈お喋りを通して自身の生活を振り返ってもらうよう導く〉ことで，介護予防の必要性を促し，〈高齢者の意向を確認しながら少しずつ介入する〉ことで《高齢者の介護予防に向けた行動変容へ導く》行動をしていた．地域に向けては，介護予防を発信したり，〈地域住民に介護予防に向けた地域づくりの先行事例や効果をふまえてわかりやすく説明する〉ことで《介護予防に向けた地域づくりを牽引する》行動をしていた．

（5）【ケアチームのコーディネーション】

このコアカテゴリーは，高齢者を支援するケアチームの連携が高まるようにコーディネートする行動で，2 つのカテゴリーから構成された．先ず，ケアチームを構築するために〈培ったネットワークを活用して，支援方法を模索する〉ことをしながら，《支援に関わってもらえ

る人・地域の社会資源を探し繋げる》ことをしていた．その後，〈支援者・関係機関のそれぞれの立場を理解して役割を調整する〉ことをしていた．研究参加者の*「病院と在宅のギャップを埋めるところに対しては，医療知識がある者としての役割かなと思います．(I 氏)」*との語りのように，医療と介護のつなぎ役として〈医療職と福祉職との連携をファシリテートする〉こともしていた．そうすることで，《高齢者の支援者らの連携が高まるように調整する》行動をしていた．

（6）【地域で実施できる事業・資源の創設】

このコアカテゴリーは，運動教室やサロン等を地域で運営実施できる事業を創設する行動で，2 つのカテゴリーから構成された．〈事業の素案を地域の住民や関係機関に相談して意向を聞く〉ことを行い，ネットワークを活かして運営の実行メンバーをつくり事業を立ち上げていた．事業運営に向けては，研究参加者の*「(住民の）やりたいこととモチベーションでどこまでできるかというところをみながら・・・(C 氏)」*の語りのように，〈事業に関わる住民や関係機関の運営状況を見極め，継続できるようにサポートする〉ことで，《地域でできる事業を企画し，立ち上げ・運営を推進する》ことをしていた．また，〈今ある地域の事業を評価して，解決できていない部分に対して，新たな事業の開発の可能性を考える〉ことで，《地域にない資源に気付き，新たな資源開発を展望する》行動もしていた．

（7）【地域のケアシステムづくり】

このコアカテゴリーは，高齢者のニーズを地域で支援する姿を目指して，地域の課題を地域で解決できるように働きかける行動で，3 つのカテゴリーから構成された．看護師は，〈地

域課題に対する地域の状況・情報を収集して、地域ケア会議などの場で住民に説明する〉ことや，〈気になっている地域の課題・心配事を積極的に会議の場で提案する〉ことで，先ず《住民や地域の関係者と解決すべき地域課題を共有する》ことをしていた．研究参加者の「*私こういうのやってんだけど地域でできないだろうか，という相談が入って．認知症カフェがあるからお願いしたい，ってマッチングしたの．（F氏）*」との語りのように，〈培ったネットワークを活用して，地域のニーズとニーズをマッチングさせる〉など《地域の課題解決策を提案する》行動をしていた．また，研究参加者の「*地域に資源があっても，誰も知らないと使ってもらえないので，それを一覧表にして民協とか地域の集まりの時に紹介してるんです（C氏）*」の語りのように〈地域の社会資源を地域で活用できるように働きかける〉ことや〈住民や関係者との連携会議を定期的に開催して，話し合える機会をつくる〉などをして，《住民や関係者・関係機関との支援の連携が地域で高まるように働きかける》行動をしていた．

（8）【地域包括支援センター職種間のチームワーク】

このコアカテゴリーは，地域包括支援センター内のチームワークを高めるための行動で，3つのカテゴリーから構成された．看護師は，〈日常的に報告・連絡・相談を行い，常に地域包括支援センターの職種間で情報共有をする〉ことを行い，〈常に事例の支援方法を地域包括支援センターの職種間で話し合って組織で支援をする〉行動をしていた．そうすることで，《地域包括支援センターのどの職種でも対応できる体制をつくる》ことを重視していた．また，〈地域包括支援センターの職種間で専門的知識や技術を教え合う〉ことで《地域包括支援センターの各専門性を効果的に活用する》ことをしていた．一方で，《地域包括支援センター職

種間の関係性を良好にする》ために，看護師の専門性を表出して活動する時と，他職種との関係性を鑑み看護師の表出を抑える時があるなど〈看護師の役割意識の表出を調整する〉行動も抽出された．

（9）【専門性を高めるための自己研鑽】

このコアカテゴリーは，地域包括支援センターの機能役割に即した支援ができるように自身をマネジメントする行動で，3 つのカテゴリーから構成された．《地域包括支援センター職員としての支援技術を高める》ために，〈話し合いの場で活用できるファシリテーションスキルを高める〉ことや，〈相手に理解してもらえる様な伝え方ができるように努力する〉ことをしていた．〈近隣市町村の取組み，国・県からの情報，活動報告などの情報を収集する〉などして，《業務遂行に必要な知識・情報の更新を図る》行動をしていた．研究参加者から*「いろんな人と繋がることが楽しい（D 氏）」*との言葉も聞かれ，《住民と共に活動する楽しさを感じる》気持ちを持っていた．

（10）【効果的な支援を実践するための業務マネジメント】

このコアカテゴリーは，地域包括支援センターとして効果的な実践をするために業務をマネジメントする行動で，2 つのカテゴリーから構成された．看護師は，《計画的に業務を遂行する》ために，質を担保するための業務整理をしたり，実施可能なことは早め早めに取り組むことをしていた．〈緊急対応が必要となった際は，素早く決断して取り組む〉ことが求められるため，《素早く判断して、優先順位を考えて業務を遂行する》意識を持っていた．

考察

地域包括支援センター看護師の実践行動の特徴

本研究では，10 コアカテゴリーの【介護予防に向けたアセスメント】,【信頼関係を育むコミュニケーション】,【ネットワークづくり】,【介護予防の牽引】,【ケアチームのコーディネーション】,【地域で実践できる事業・資源の創設】,【地域のケアシステムづくり】は，高齢者および地域を介護予防支援する行動として,【地域包括支援センター職種間のチームワーク】,【専門性を高めるための自己研鑽】,【効果的な実践をするための業務マネジメント】は，地域包括支援センター組織の一員として対応する行動として構成されると考えられた．また，地域包括支援センター看護師の実践行動には，3 つの特徴が考えられた．

1 つ目は，病院の臨床経験に基づいたスキルを生かして，地域の住民や関係者らの連携をサポートしていたことである．本結果では，高齢者の医療の必要性や病状に応じた医療的介入のタイミングの判断,〈医療職と福祉職との連携をファシリテートする〉行動が示された．地域包括ケアシステムの構築に向けて，在宅医療・介護の連携や認知症施策の推進が取り組まれている [1] が，地域包括支援センターの看護師には，生命への適切な対応力や医療と介護のつなぎ役としての役割が期待できると考えられる．

2 つ目は，個別支援の視点と地域づくりの視点を持って実践していることである．この結果は，地域包括支援センター保健師の活動と類似していた [9-12]．本結果では，個別支援などの日常業務で培ったネットワークを活用して《支援に関わってもらえる人・地域の社会資源を探し繋げる》支援をしていた．看護師は，民生委員，近隣住民や関係機関などあらゆる地域

の人々と連携し，地域の資源を模索し活用できるよう調整しながら支援をしており，地域の状況に合わせて，地域の人と共に支援する行動の基盤が，個別と地域の両者の視点を用いて支援をする背景になっていると考えられる．

3 つ目は，個別支援の支援方法を模索する中で繋げた支援を地域で活用できるように広めることで，地域のケアシステムづくりを目指していることである．本結果では，高齢者を支援する中で，住民や関係機関と情報交換をしながら，高齢者を支援する仲間を増やし，その事例を積み重ねて，地域の【ネットワークづくり】をしていた．この培ったネットワークを活用し，地域のニーズとニーズをマッチングさせたり，地域の資源を住民や支援者らが活用するように働きかけたりしていた．三輪（2022）は，地域ケアにおける看護師は行政保健師とは異なる視点で健康課題を発見し地域活動を生み出す可能性を述べている[14]．本結果では，個別事例の健康課題に対する支援方法を模索する中で，新たな地域の資源を発掘し支援につなげることで，地域の資源を開発するきっかけとしていた．また，つながった地域の資源を，他の事例や他の支援関係者も活用できるように広めることで、地域の資源を地域で活用できるように働きかけていた．これらの地域包括支援センター看護師の行動は，地域のケアシステムを構築する方法のひとつとなる可能性が推察された．

本研究の限界と今後の課題

本研究の限界として，第 1 に研究参加者の偏りである．1 県内の高齢化率が全国平均より低い中小都市部の 7 市 9 施設で勤務している 10 人と，対象地域と対象者数が限定されていることが挙げられる．全国の地域包括支援センターの実態調査によると，人口 50 万人以上の

大都市は，総合相談業務の時間が長く，人口 5 万人未満の市町村は認知症支援や一般介護予防支援の業務時間が長いと報告されており[13]，人口規模や高齢化率，地域性等によって業務の内容が異なり，データに影響している可能性がある．第 2 に研究方法の限界である．本研究では，看護師の行動を幅広く探索することを重視したため，質的記述的方法を行った．成果として，看護師の具体的な実践行動が抽出され特徴的な行動が示唆されたことは，一定の意義があると考える．一方で、質的分析による結果への影響の可能性は否めない．今後は，Delphi 法を含めた大規模な内容妥当性の検証をする必要があると考える．

謝辞

本研究にご協力くださいました地域包括支援センター看護師の皆様に深謝いたします．本研究は，JSPS 科研費 21K11041 の助成を受けて実施した．

利益相反

開示すべき COI はない．

【文献】

1. Health, Labour and Welfare Statistics Association. Kokumineisei no doukou (National health trends). Tokyo: Health, Labour and Welfare Statics Association; 2024. 225-7 (in Japanese).
2. Mitsubishi sogo kenkyujo. Chiikihokatsushiensenta ni okeru gyomujittai ni kansuru chosakenkyujigyo hokokusho ; 2015 (in Japanese). <https://pubpjt.mri.co.jp/pjt_related/roujinhoken/jql43u00000001m5-att/h26_03.pdf>

(Accessed January 14, 2025)

3. Sakurai M, Saeki K, Takahashi Y, Sido K, Kanbara R, Ohotomo Y, Nagai M, Miyake K. Hokkaido no Chiiikihokatsushiencenta ni kinmusuru hokenshi no shokugyosei sutoresu to shokumu manzokudo (Occupational stress and job satisfaction of public health nurses working at Community General Support Centers in Hokkaido). Hokkaido Journal of Public Health 2011; 24: 49-56 (in Japanese).
4. Spencer LM, Spencer SM. Competence at work : models for superior performance. New York: Wiley; 1993: 11-9.
5. Shiomi M, Okamoto R, Iwamoto A. Development of competency measurement concerning the creation of projects/ social resources for public health nurses: Investigation of reliability and validity. Japanese journal of public health 2009; 56: 391-401 (in Japanese).
6. Iwamoto S, Okamoto R, Shiomi M. Development and evaluation of the reliability and validity of a scale for basic actions relevant to public health. Japanese journal of public health 2008; 55: 629-39 (in Japanese).
7. Inoue H. Management competency of nurse manager. Journal of Kochi Women's University Academy of Nursing 2014; 40: 109-16 (in Japanese).
8. Iwamoto M, Utsumi T, Hosohara M, Koju K. Identification of competency elements required for risk sensitivity. Journal of Kagawa Prefectural University of Health

Sciences 2014; 5: 15-22 (in Japanese).

9. Kawamoto A, Taguchi A, Kuwahara Y, Matsunaga A, Iwasaki R, Murashima S. Contents of Individual Support Implemented by Cooperation between Neighbors and Public Health Nurses at Comprehensive Community Support Centers in Japan. Journal of Japan Academy of Community Health Nursing 2012; 15: 109-18 (in Japanese).

10. Ohtaka N, Sasaki A, Tanuma T, Morita K. A view of public health nurses working Community General Support Center on care prevention in community activities for elder people. Journal of the Ochanomizu Association for Academic Nursing 2012; 6: 70-80 (in Japanese).

11. Yoshida R, Izumi H, Katakura Y, Namikawa K. The Development of Guidelines in Public Health Nursing Practice for the Promotion of Preventive Long-term Care Systems. Journal of Japan Academy of Community Health Nursing 2012; 14: 5-13 (in Japanese).

12. Miyamoto M, Yanagisawa S. Competencies of public health nurses who work at a Community general support center. Journal of Japan academy of nursing for home care 2023; 11: 57-67 (in Japanese).

13. Mitsubishi UFJ Research & Consulting. Chiikihokatsushiensenta no gyoumuzittai ni kansuru chosakenkyujigyo hokokusho ; 2019 (in Japanese).

<https://www.murc.jp/wp-content/uploads/2019/04/koukai_190410_7.pdf> (Accessed May 28, 2025)

14. Miwa K, Kono A. Community Empowerment Processes and Competencies of Nurses in Community-based Care. Journal of Japan Academy of Nursing Science 2022; 42: 899-907 (in Japanese).

Table 1. Overview of study participants and their affiliated institutions

	A	B	C	D	E	F	G	H	I	J
Age	50s	60s	50s	50s	50s	50s	60s	40s	50s	50s
Years of experience as a nurse	15	20	34	20	30	36	50	20	21	30
Mean ± Standard deviation	25.2 ± 12.5									
Years of experience at community general support center	12	11	17	10	13	15	14	9	11	16
Mean ± Standard deviation	12.8 ± 2.5									
Facility management	Commissioned management	Commissioned management	Commissioned management	Commissioned management	Commissioned management	Commissioned management	Commissioned management	Commissioned management	Commissioned management	Commissioned management
Municipality	City Z	City Y	City X	City W	City X	City X	City V	City V	City U	City T
Population size of municipality	Medium city[2]	Core city[1]	Small city[3]	Medium city[2]	Small city[3]	Small city[3]	Medium city[2]	Medium city[2]	Small city[3]	Medium city[2]
Aging rate of municipality	Approx. 25%	Approx. 24%	Approx. 27%	Approx. 20%	Approx. 27%	Approx. 27%	Approx. 26%	Approx. 26%	Approx. 26%	Approx. 27%

1) Core city: Population over 200,000

2) Medium city: Population over 100,000

3) Small city: Population below 100,000

Table 2. Categories for the practical actions of nurses at community general support center

Core category	Category
Assessment for care prevention	observe and listen to their actual life circumstances from a wide range of perspectives to gain understanding of the actual living conditions and assess the lifestyle desired by the older adult and their families from the perspective of nursing care prevention
	Assess local issues and needs based on local conditions
	Have a vision for feasible regional development that makes use of the characteristics of the region
Communication that builds trust	Listen carefully to the older adult and residents and understand them
	Raising awareness of the community general support center
	Promoting mutual understanding through dialogue
Networking	Raising awareness of the community general support center and creating opportunities for connections
	Creating routes to connect with information
	Building a network by accumulating routes to connect with information
Promotion of nursing care prevention	Leading older adults to adopt behavioral changes to prevent the need for nursing care
	Promoting communities for nursing care prevention
Coordination of the care team	Find and connect with people who can provide support and local social resources
	Coordinate to increase cooperation among those who support older adults
Creation of projects and resources that can be locally implemented	Plan local projects and promote their launch and operation
	Discovering resources not locally available and looking ahead to developing new resources
Creation of a local care system	Share local issues that need to be resolved with residents and people connected to the area
	Proposing solutions to local issues
	Work to increase cooperation between residents, related parties, and relevant organizations in the region
Teamwork among professions at the community general support center	Create a system that can handle any type of job at the community general support center
	Effectively utilize the expertise of each community general support center
	Improve relationships between professions at the community general support center
Self-improvement to improve one's expertise	Improving support skills as a staff member at the community general support center
	Update the knowledge and information necessary to carry out works
	Enjoying working with residents
Operational management for implementing effective support	Carry out the works in a planned manner
	Make quick decisions and prioritize works
